# Time course of bone metabolism at the residual ridge beneath dentures observed using ^18^F-fluoride positron emission computerized-tomography/computed tomography (PET/CT)

**DOI:** 10.1007/s12149-012-0648-6

**Published:** 2012-08-18

**Authors:** Hanako Suenaga, Masayoshi Yokoyama, Keiichiro Yamaguchi, Keiichi Sasaki

**Affiliations:** 1 Division of Advanced Prosthetic Dentistry, Tohoku University Graduate School of Dentistry, 4-1 Seiryo-machi, Aoba-ku, Sendai, 980-8575 Japan; 2 Department of Radiology, Sendai Kousei Hospital, 4-5 Hirose-machi, Aoba-ku, Sendai, 980-0873 Japan

**Keywords:** Bone metabolism, Denture, Mechanobiology, ^18^F-fluoride PET

## Abstract

**Objective:**

Excessive pressure due to wearing mal-adapting dentures is well known to cause residual bone resorption beneath the denture. X-rays have been commonly utilized to evaluate the changes in the bone beneath the denture. However, X-ray images merely detect bone density and relatively large changes in the bone shape and structure, whereas nuclear medicine imaging can detect functional changes, which occur prior to structural changes. This article aimed to describe the time course of the bone metabolism at the residual ridge beneath the denture following denture use by ^18^F-fluoride positron emission computerized-tomography (PET)/computed tomography (CT) scanning.

**Methods:**

Three subjects, who had a free-end edentulous mandible, were treated with a denture replacing the edentulous region of the dental arch. The metabolic changes in the residual bone beneath the denture were assessed by ^18^F-fluoride PET/CT imaging. ^18^F-fluoride PET/CT scanning was performed at baseline, and 4–6 and 13 weeks after denture use. A volume of interest (VOI) was placed on their mandibles at the edentulous region beneath the denture on the PET/CT image. CT value and mean standardized uptake value (SUV) of the VOI were calculated. The difference in the time variation between the CT value and SUV was analyzed.

**Results:**

The adaptation of the denture base to the residual ridge was successful, and there was no trouble such as pain at the residual ridge beneath the denture. The SUVs of each VOI significantly increased at 4–6 weeks after denture use and then decreased at 13 weeks in all three subjects (*P* < 0.05; two-way ANOVA, Dunnett test). On the other hand, the CT images showed no obvious changes in the bone shape or structure beneath the dentures, and the CT values of each VOI remained static after denture use in all three subjects.

**Conclusions:**

This study indicates that in the present first-time removable partial denture (RPD) users, wearing of a well-adapted RPD initially increased bone metabolism beneath the denture and then decreased it at around 13 weeks after RPD use without any bone structural changes detectable by clinical X-rays. These metabolic changes are a mechanobiological reaction to the pressure induced by RPD use.

## Introduction

As indicated by Wolff’s law [1], bone is remodeled to meet its mechanical demands, suggesting that mechanical forces are among the most potent factors that influence bone formation and resorption. In contrast, the structure and volume of the bone are maintained by the tireless bone metabolism so that bone deformation is considered to be a consequence of the bone metabolic changes. Therefore, it is considered that the mechanical load induces metabolic changes in bone at the beginning as a mechanobiological reaction.

Excessive pressure due to wearing mal-adapting dentures is well known to cause bone resorption beneath the denture [2]. X-rays have been commonly utilized to evaluate the changes in the bone beneath the denture. However, X-ray images merely detect bone density and relatively large changes in the bone shape and structure, whereas a nuclear-based procedure can detect functional changes in the bone that occur prior to structural changes. In particular, ^18^F-fluoride positron emission computerized-tomography (PET) has received much attention in detecting bone diseases such as bone metastases because of its high image quality [3–5]. The functional imaging technique of ^18^F-fluoride PET allows a quantitative assessment of bone metabolism at specific sites of the skeleton in man [6–16], suggesting that the technique could be used to quantify the magnitude of the localized response to pressure induced by wearing dentures. In addition, ^18^F-fluoride PET should be useful for serial scanning to document the time course of the skeletal response after loading, because of the short half-life of ^18^F-fluoride (<2 h).

This study aimed to describe the time course of the bone metabolism at the residual ridge beneath the denture following removable partial denture (RPD) use by ^18^F-fluoride PET/computed tomography (CT) scanning.

## Materials and methods

### Subjects

**Fig. 1:**
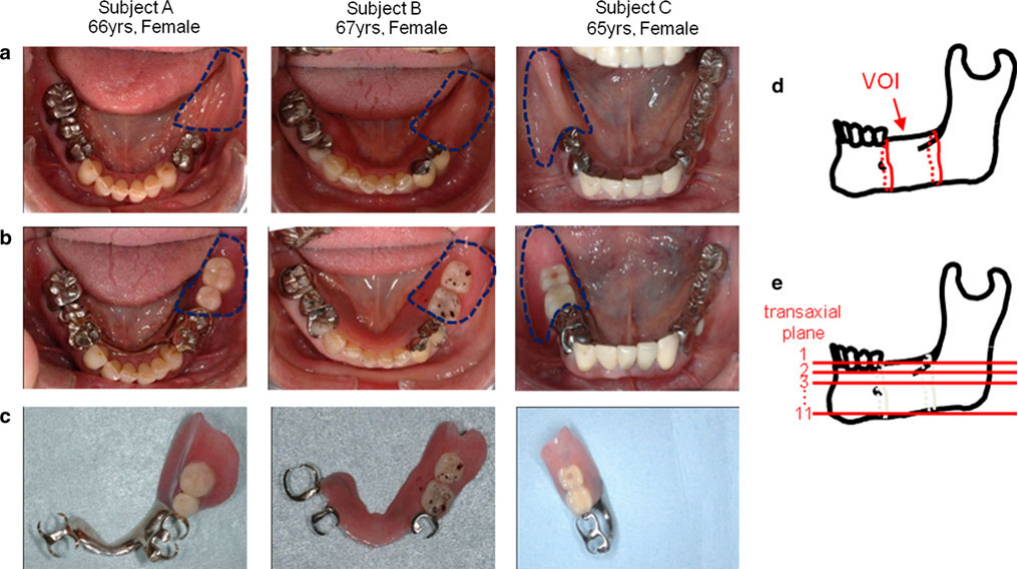
Subjects and definition of VOI and ROI. **a** Occlusal views of the mandibles. *Dashed line* indicates edentulous region of dental arch; **b** occlusal views of the mandibles with the RPDs replacing edentulous regions indicated by *dashed lines*; **c** RPDs; **d** definition of VOI; **e** definition of transaxial plane number. ROI: edentulous regions of mandibles in transaxial planes

A 66-year-old woman (subject A), a 67-year-old woman (subject B), and a 65-year-old woman (subject C) without abnormal bone metabolism consulted the Advanced Prosthetic Dentistry of Tohoku University Hospital. Their chief complaint was masticatory dysfunction on the edentulous region of the dental arch. Subject A had lost her mandibular left molars more than 8 years, subject B had lost her mandibular left molars and second premolar more than 1 year, and subject C had lost her mandibular right molars more than 1 year before presenting to our hospital. They had no experience with mandibular dentures (Fig. 1a). All three subjects chose unilateral distal extension RPD replacing the edentulous region of the dental arch (Fig. 1b). We fabricated a platinum metal base RPD for subject A, a resin base RPD for subject B, and a cobalt chrome metal base RPD for subject C (Fig. 1c). The adaptation of the denture base to the residual ridge was checked using white silicon (Fit checker, GC Co., Tokyo, Japan) at baseline and 13 weeks after RPD use. Occlusal surfaces of the artificial teeth were adjusted to distribute the symmetrical occlusal contacts within the dental arch. A day after the first use of the RPD, a postinsertion adjustment was made.

### ^18^F-fluoride PET/CT imaging

The metabolic changes in the residual bone beneath the RPD were assessed using an ^18^F-fluoride PET/CT imaging scanner (Discovery ST Elite, GE Healthcare Japan Co., Tokyo, Japan). ^18^F-fluoride PET was performed at baseline, and 6 and 13 weeks after RPD use for subjects A and C, and at baseline, and 4 and 13 weeks for subject B. Research protocols for this study were approved by the research ethics committee at both the Tohoku University Graduate School of Dentistry and Sendai Kousei Hospital. Signed consent forms were obtained after full explanation of the procedures from the three subjects. The emission scans in the three-dimensional acquisition mode with spatial resolutions of 2.0, 2.0, and 3.27 mm in the radial, tangential, and axial directions (Table 1) were started 75 min after intravenous injection of 37 MBq ^18^F-fluoride. Subjects were positioned supine with the jaw bone in the field of view (FOV). The scan time of the jaw bone was extended from the regular 3 to 20 min to obtain clear PET images even with a low ^18^F-fluoride dosage (1/5 the regular dosage per scan). In all cases, low-dose CT was performed with exposure parameters of 120 kV, tube current 15 mA, and 3.75 mm slice thickness.

**Table 1:** Scanning condition

	Field of view (mm)	Matrix	Slice thickness (mm)
PET	256	128 × 128	3.27
CT	256	256 × 256	3.75

### Data analyses

The PET and the CT data were processed and fused using medical image viewer software (EV Insite R, PSP Co., Tokyo, Japan) to identify the anatomical location. Subsequently, the volumes of interest (VOIs) were placed on their mandibles at the edentulous regions beneath the RPDs, which included the mandibular bone from the distal end of the direct abutment to the distal border of the retromolar pad (Fig. 1d). Furthermore, the regions of interest (ROIs) were placed at the edentulous regions of the mandibles beneath the RPDs in the transaxial planes numbered from superior, 1, to inferior, 11 (Fig. 1e). CT values and the mean standardized uptake values (SUVs) were measured for each ROI. Subjects A, B, and C had 7, 11, and 9 ROIs, respectively. The SUV of VOI, defined as the average value of SUV of all ROIs in each subject, was calculated. The CT value of VOI was also calculated in the same way. In each subject, the differences between the serial CT values of VOI and between the serial SUVs of VOI were tested using two-way analysis of variance (ANOVA) and Dunnett test for post hoc analyses (Dr. SPSS II for Windows, SPSS Inc., Chicago, IL, USA). The SUV of ROI was analyzed to examine the effect of time and distance from the RPD.

## Results

The adaptation of the denture base to the residual ridge was successful at both baseline and 13 weeks after RPD use. An interview and questionnaire revealed that the masticatory function was improved after RPD use and that there was no trouble such as pain at the residual ridge beneath the denture in any of the three subjects.

**Fig. 2:**
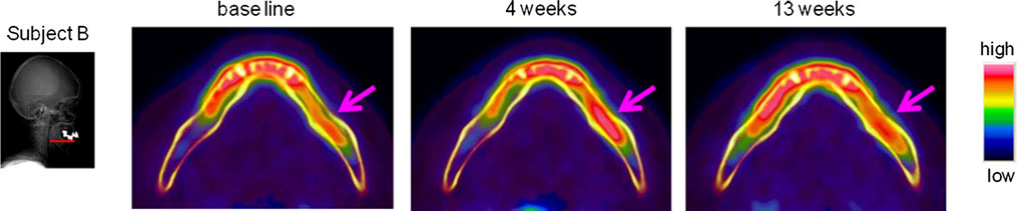
Transaxial plane images, indicated by a *line* in *left diagram*, of accumulation of ^18^F-fluoride at baseline, and 4 and 13 weeks after RPD use. *Arrow* indicates region of residual ridge beneath the RPD

**Fig. 3:**
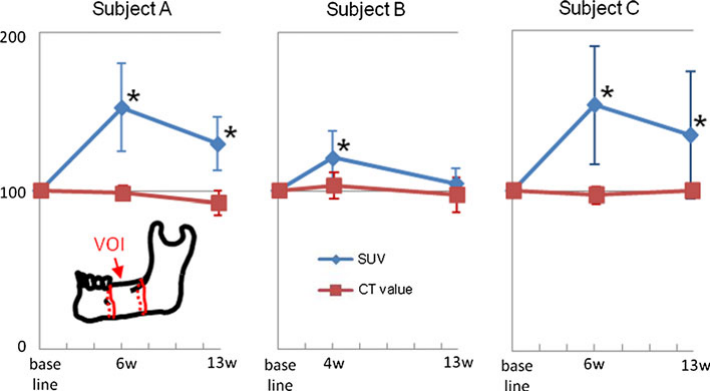
Percentage changes in the SUVs and the CT values of VOIs. *P < 0.05; two-way ANOVA, Dunnett test

**Fig. 4:**
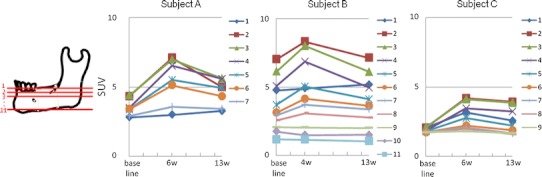
Changes in the SUVs of ROIs

The SUVs of each VOI were significantly increased at 4–6 weeks after RPD use and then decreased at 13 weeks in all three subjects (*P* < 0.05; two-way ANOVA, Dunnett test) (Figs. 2, 3). The CT images, on the other hand, showed no obvious changes in the bone shape or structure beneath the RPDs, and the CT values of each VOI remained static after the RPD use in all three subjects (Fig. 3). Figure 4 showed serial changes in the SUVs of each ROI during treatment with RPDs. Except for transaxial plane 1, the SUVs of ROIs tended to increase as the number of the transaxial planes decreased, or in other words, as the distance from the RPD decreased. This tendency became pronounced after RPD use. Moreover, the SUV of ROIs in transaxial planes 2–8 tended to increase after RPD use, whereas those in planes 9–11 remained static.

## Discussion

Radiographic methods have been predominantly used not only in clinical practice but also in human studies of residual ridge resorption. In these studies, X-ray images were usually taken before denture use and a few years afterwards [17–25]. In our previous report, we established a scanning procedure to detect bone metabolic changes beneath the RPD using ^18^F-fluoride PET/CT [26]. Clear PET images were successfully obtained with low-level radiation exposure by extending the scan time to 20 min, which enables us to scan several times per subject. In this study, we applied this procedure to three subjects and examined the detailed dynamic and longitudinal processes in the biologic responses of residual bone beneath the denture immediately after the first RPD use.

In the present investigation, the SUVs of VOIs and ROIs in transaxial planes 2–8 increased after RPD use. This may represent not increased blood flow as an inflammatory reaction but rather increased bone metabolism as a mechanobiological reaction to the pressure due to RPD use based on the following: (1) No pain or inflammation is reported in the denture supporting tissue in clinical practice. (2) If it is inflammatory reaction, the response of the periosteum, which is highly vascular, would be marked. Actually the SUVs in transaxial plane 1 including the periosteum were not so high. (3) In the case of inflammatory reaction, blood flow increases for fewer than 2–3 weeks, whereas the SUVs in the present cases increased at 4–6 weeks.

Our previous animal studies of bone metabolism beneath the denture base and around loaded implants using bone scintigraphy [27, 28] showed that relatively low mechanical stress initially increased bone metabolism to the peak level, which declined to the baseline level with time. In this study, the SUV changes revealed a similar trend to that in our previous studies, and the SUV initially increased at 4–6 weeks after RPD use and then decreased at 13 weeks in all three subjects. The adaptation of the denture base to the residual ridge was successful, and no pain or inflammation was reported in the denture supporting tissue, implying that this change may be temporary and may have been related to the process of bone adaptation to the RPD. Furthermore, our previous animal study also indicated that the change pattern of bone metabolism during 6 weeks after loading depended on the magnitude of the load [28]. Thus, excessive stress may produce a different SUV change pattern in human case studies during 13 weeks or so.

Low SUVs of ROIs in transaxial plane 1 were most likely due to the fact that transaxial plane 1 contained a high level of cortical bone whereas the other transaxial planes contained mostly cancellous bone. The absence of change in the SUVs of ROIs in transaxial planes 9–11 may be caused by mechanical loading due to RPD use not being transmitted to those planes, which were far away from the RPDs.

In conclusion, this study indicates that in first-time RPD users, wearing of a well-adapted RPD initially increases bone metabolism beneath the denture after which it decreases at around 13 weeks after RPD use without any bone structural changes detectable by clinical X-rays. This metabolic change is a mechanobiological reaction to the pressure induced by RPD use.

## References

[1] Wolff J (1986). The law of bone remodeling.

[2] Carlsson GE (2004). Responses of jawbone to pressure. Gerodontology.

[3] Yen RF, Chen CY, Cheng MF, Wu YW, Shiau YC, Wu K (2010). The diagnostic and prognostic effectiveness of F-18 sodium fluoride PET-CT in detecting bone metastases for hepatocellular carcinoma patients. Radiology.

[4] Krüger S, Buck AK, Mottaghy FM, Hasenkamp E, Pauls S, Schumann C (2009). Detection of bone metastases in patients with lung cancer: 99mTc-MDP planar bone scintigraphy, 18F-fluoride PET or 18F-FDG PET/CT. Eur J Nucl Med Mol Imaging.

[5] Grant FD, Fahey FH, Packard AB, Davis RT, Alavi A, Treves ST (2008). Skeletal PET with 18F-fluoride: applying new technology to an old tracer. J Nucl Med.

[6] Schirrmeister H, Guhlmann A, Elsner K, Kotzerke J, Glatting G, Rentschler M (1999). Sensitivity in detecting osseous lesions depends on anatomic localization: planar bone scintigraphy versus 18F PET. J Nucl Med.

[7] Al-Beyatti Y, Siddique M, Frost ML, Fogelman I, Blake GM. Precision of (18)F-fluoride PET skeletal kinetic studies in the assessment of bone metabolism. Osteoporos Int. 2012 [Epub ahead of print]. 10.1007/s00198-011-1889-2 22237817

[8] Siddique M, Blake GM, Frost ML, Moore AE, Puri T, Marsden PK, Fogelman I. Estimation of regional bone metabolism from whole-body (18) F-fluoride PET static images. Eur J Nucl Med Mol Imaging. 2011 (Epub ahead of print). 10.1007/s00259-011-1966-y 22065012

[9] Siddique M, Frost ML, Blake GM, Moore AE, Al-Beyatti Y, Marsden PK, Schleyer PJ, Fogelman I (2011). The precision and sensitivity of (18) F-fluoride PET for measuring regional bone metabolism: a comparison of quantification methods. J Nucl Med.

[10] Frost ML, Siddique M, Blake GM, Moore AE, Marsden PK, Schleyer PJ, Eastell R, Fogelman I. Regional bone metabolism at the lumbar spine and hip following discontinuation of alendronate and risedronate treatment in postmenopausal women. Osteoporos Int. 2011 [Epub ahead of print]. 10.1007/s00198-011-1805-9 21983795

[11] Blake GM, Siddique M, Frost ML, Moore AE, Fogelman I (2011). Radionuclide studies of bone metabolism: do bone uptake and bone plasma clearance provide equivalent measurements of bone turnover?. Bone.

[12] Frost ML, Siddique M, Blake GM, Moore AE, Schleyer PJ, Dunn JT, Somer EJ, Marsden PK, Eastell R, Fogelman I (2011). Differential effects of teriparatide on regional bone formation using (18) F-fluoride positron emission tomography. J Bone Miner Res.

[13] Waterval JJ, Van Dongen TM, Stokroos RJ, Teule JG, Kemerink GJ, Brans B, Nieman FH, Manni JJ (2011). Bone metabolic activity in hyperostosis cranialis interna measured with 18F-fluoride PET. Eur J Nucl Med Mol Imaging.

[14] Czernin J, Satyamurthy N, Schiepers C (2010). Molecular mechanisms of bone 18F-NaF deposition. J Nucl Med.

[15] Uchida K, Nakajima H, Miyazaki T, Yayama T, Kawahara H, Kobayashi S, Tsuchida T, Okazawa H, Fujibayashi Y, Baba H (2009). Effects of alendronate on bone metabolism in glucocorticoid-induced osteoporosis measured by 18F-fluoride PET: a prospective study. J Nucl Med.

[16] Hawkins RA, Choi Y, Huang SC, Hoh CK, Dahlbom M, Schiepers C, Satyamurthy N, Barrio JR, Phelps ME (1992). Evaluation of the skeletal kinetics of fluorine-18-fluoride ion with PET. J Nucl Med.

[17] López-Roldán A, Abad DS, Bertomeu IG, Castillo EG, Otaolaurruch ES (2009). Bone resorption processes in patients wearing overdentures. A 6-years retrospective study. Med Oral Patol Oral Cir Bucal.

[18] Jacobs R, van Steenberghe D, Nys M, Naert I (1993). Maxillary bone resorption in patients with mandibular implant-supported overdentures or fixed prostheses. J Prosthet Dent.

[19] Kordatzis K, Wright PS, Meijer HJ (2003). Posterior mandibular residual ridge resorption in patients with conventional dentures and implant overdentures. Int J Oral Maxillofac Implants.

[20] Sağlam AA (2002). The vertical heights of maxillary and mandibular bones in panoramic radiographs of dentate and edentulous subjects. Quintessence Int.

[21] Xie Q, Wolf J, Ainamo A (1997). Quantitative assessment of vertical heights of maxillary and mandibular bones in panoramic radiographs of elderly dentate and edentulous subjects. Acta Odontol Scand.

[22] Yüzügüllü B, Gulsahi A, Imirzalioglu P (2009). Radiomorphometric indices and their relation to alveolar bone loss in completely edentulous Turkish patients: a retrospective study. J Prosthet Dent.

[23] Wical KE, Swoope CC (1974). Studies of residual ridge resorption. I. Use of panoramic radiographs for evaluation and classification of mandibular resorption. J Prosthet Dent.

[24] Brkovic-Popovic S, Stanisic-Sinobad D, Postic SD, Djukanovic D (2008). Radiographic changes in alveolar bone height on overdenture abutments: a longitudinal study. Gerodontology.

[25] Kreisler M, Behneke N, Behneke A, Hoedt B (2003). Residual ridge resorption in the edentulous maxilla in patients with implant-supported mandibular overdentures: an 8-year retrospective study. Int J Prosthodont.

[26] Suenaga H, Yokoyama M, Yamaguchi K, Sasaki K (2012). Bone metabolism of residual ridge beneath the denture base of an RPD observed using NaF-PET/CT. J Prosthodont Res.

[27] Yokoyama M, Sasaki K, Yamaguchi K, Sasaki H, Atsumi T, Koyama S (2005). Evaluation of bone metabolism of alveolar bone beneath the denture base and temporomandibular joint. Int Congr Ser.

[28] Sasaki H, Koyama S, Yokoyama M, Yamaguchi K, Itoh M, Sasaki K (2008). Bone metabolic activity around dental implants under loading observed using bone scintigraphy. Int J Oral Maxillofac Implants.

